# Research on the pathological mechanism of rectal adenocarcinoma based on DNA methylation

**DOI:** 10.1097/MD.0000000000032763

**Published:** 2023-01-27

**Authors:** Xiaoqiang Pan, Xingcheng Yi, Maozhuo Lan, Xiaoyun Su, Fang Zhou, Wei Wu

**Affiliations:** a Department of Neurosurgery, The First Hospital of Jilin University, Changchun, Jilin, China; b Laboratory of Cancer Precision Medicine, The First Hospital of Jilin University, Changchun, Jilin, China; c School of Pharmaceutical Sciences, Jilin University, Changchun, China; d Equipment Division, Zhijiang People’s Hospital, Yichang, Hubei, China.

**Keywords:** canonical correlation analysis (CCA), DNA methylation, READ, weighted gene regulatory network analysis

## Abstract

Colorectal cancer is one of the 3 most common cancers worldwide. In this study, a weighted network-based analysis method was proposed to explore the pathological mechanisms and prognostic targets of rectal adenocarcinoma (READ) at the deoxyribonucleic acid (DNA) methylation level.

In this study, we downloaded clinical information and DNA methylation data from The Cancer Genome Atlas database. Differentially methylated gene analysis was used to identify the differential methylated genes in READ. Canonical correlation analysis was used to construct the weighted gene regulatory network for READ. Multilevel analysis and association analyses between gene modules and clinical information were used to mine key modules related to tumor metastasis evaluation. Genetic significance analysis was used to identify methylation sites in key modules. Finally, the importance of these methylation sites was confirmed using survival analysis.

DNA methylation datasets from 90 cancer tissue samples and 6 paracancerous tissue samples were selected. A weighted gene regulatory network was constructed, and a multilevel algorithm was used to divide the gene co-expression network into 20 modules. From gene ontology enrichment analysis, characteristic M was related to biological processes such as the chemotaxis of fibroblast growth factors and the activation and regulation of immune cells etc and characteristic N was associated with the regulation of cytoskeleton formation, mainly microtubules and flagella, regulation of synapses, and regulation of cell mitosis. Based on the results of survival analysis, 7 key methylation sites were found closely correlated to the survival rate of READ, such as cg04441191 (microtubule-associated protein 4 [MAP4]), cg05658717 (KSR2), cg09622330 (GRIN2A), cg10698404 (YWHAG), cg17047993 (SPAG9), cg24504843 (CEP135), and cg24531267 (CEP250). Mutational and transcriptomic level studies revealed significant differences in DNA methylation, single nucleotide polymorphism, and transcript levels between YWHAG and MAP4 in normal tissues compared to tumor tissues, and differential expression of the 2 proteins in immunohistochemistry. Therefore, potential targeting drugs were screened for these 2 proteins for molecular docking, and artenimol was found to bind to MAP4 protein and 27-hydroxycholesterol to YWHAG.

Our study found that key methylation sites played an important role in tumor metastasis and were associated with the prognosis of READ. Mutations and methylation may jointly regulate the transcription and translation of related genes, which in turn affect cancer progression. This may provide some new potential therapeutic targets and thoughts for the prognosis of READ.

## 1. Introduction

Colorectal cancer (CRC) is one of the 3 most common cancers around the world.^[1]^ Recently, many studies have shown that many genes’ significant changes in expression levels and related biological process responses affect development and prognostic survival of rectal adenocarcinoma (READ). Chao et al analyzed READ samples (GSE35452), and found that the expression level of DSG3 is negatively correlated to poorer tumor regression and had an independent negative impact on disease-specific survival, local recurrence-free survival, and metastasis-free survival of READ.^[2]^ Millino et al found out that ABCC2 and 6 micro ribonucleic acids such as miR-7 and miR-182 were used as preoperative radiochemotherapy response targets for locally advanced rectal cancer.^[3]^ Nfonsam et al pointed out that MAPK signaling pathway was significantly out of control in divergent early-onset rectal cancer, and so was PI3K-AKT signaling pathway in late-onset rectal cancer.^[4]^ Horvat et al indicated that tumor location of READ, CRM status and lymph node metastasis was significantly related to gene APC and RASA1 mutations, ATM mutations and BRCA2 mutations respectively.^[5]^ Wu et al found out that TIPE2 could regulate the proliferation, migration and invasion of READ cells via Wnt/β-Catenin and transforming growth factor-β/Smad2/3 signaling pathways.^[6]^ In so many current studies, research on READ were mainly focused on the genes and signaling pathways related to tumor proliferation, migration and invasion at transcriptome level.

Deoxyribonucleic acid (DNA) methylation represents a basic epigenetic modification that can regulate chromatin structure and gene transcription. Abnormal methylation patterns affect the disease phenotype, such as cancer, degenerative diseases and aging etc.^[7]^ In recent years, molecular typing or pathological mechanism exploring of diseases at gene DNA methylation level has become a hotspot in epigenetics research. Westerman et al used WGCNA and Comb-p algorithm to calculate methylation regions and modules of cardiovascular diseases, and found out that methylation of SLC9A1, SLC1A5, and TNRC6C were closely related to the risk of cardiovascular diseases.^[8]^ However, the construction of a methylation co-expression network based on methylation sites mostly will lead to excessive network density and reduce the accuracy of module division. Therefore, the application of the WGCNA algorithm to methylation data is not very suitable. Moreover, the methylation level of a single methylation site cannot represent the overall methylation status.

A weighted network-based analysis method was proposed to explore the pathological mechanism and prognostic targets of READ at the DNA methylation level. First of all, a DNA methylation dataset of READ cancer tissues and normal tissues was downloaded from The Cancer Genome Atlas.^[9]^ Then, an appropriate threshold was chosen to identify differentially methylated genes (DMGs). Furthermore, the weighted gene regulatory network was constructed by canonical correlation analysis (CCA), and multilevel algorithm was used to identify gene modules. Later, we associated gene modules with clinical indicators event, age, M, N, and T, and 2 key gene modules were found closely related to tumor metastasis. At this end, we used the MM algorithm to identify key methylation sites in key gene modules and dig out the key nodes of prognostic survival by subsistence analysis. Next, we investigated the mutation, transcription, and expression of methylation site-related genes, identified 2 core genes, microtubule-associated protein 4 (MAP4) and YWHAG, and screened small molecule drugs against these 2 proteins for molecular docking to provide ideas for the mechanism of colorectal carcinogenesis as well as therapeutic targets.

## 2. Materials and Methods

### 2.1. Data collection and data processing

DNA methylation data and clinical data were downloaded from The Cancer Genome Atlas (https://www.cancer.gov/), including 103 issue samples including 97 READ samples and 6 adjacent tissue samples. The intersection samples were screened between methylated samples and clinical samples. And then, the outliers from cancer tissue DNA methylation data set were removed by hierarchical clustering with R function hclust() in the stats package (v3.6.1). At last, The DNA methylation sites with 0 *β* value were removed.

### 2.2. Identification of DMGs

In this study, we first found out whether there is a batch effect in the dataset by using the edgeR package (v3.6.1). After, we identified differentially methylated sites by using R function champ.DMP() in the ChAMP package (v3.6.1). And then, DMGs were defined as the genes corresponding to differentially methylated sites. Among them, the thresholds were |log(Foldchange)| > |log(0.235)| and *P* value <.05.

### 2.3. Construction of weighted gene regulatory network and identification of gene modules

Gene regulatory network is an interaction network constructed by the real gene and regulatory relationship between genes. In this study, standard gene regulatory network was downloaded from Wang, E. (2014). Human Signaling Network (http://www.bri.nrc.ca/wang/cancerMap/HumanSignalingNetwork_v6.csv). Next, we screened all regulatory relationships contained DMGs in the standard gene regulatory network, and then the network with the largest number of genes was used for further analysis.

In this study, principal component analysis (PCA) algorithm was applied to realize the feature reduction of all the sites contained in each gene in the gene regulatory network. To ensure the completeness of DNA methylation information of each gene, we defined the correlation of all principal components between the 2 genes calculated by the CCA algorithm as the weight of the network edge and constructed a weighted gene regulation network. Among them, PCA algorithm was implemented using R function prcomp() in the stats R package (v3.6.1). CCA algorithm was implemented using R function cancor() in the stats R package (v3.6.1).

Finally, we used R function multilevel.community()^[10]^ in the igraph R package (v3.6.1) to realize the gene module identification of the weighted gene regulatory network.

### 2.4. Association analysis of gene modules and clinical information

To explore the association between gene modules and clinical phenotypes, we performed R function champ.DMP() in the ChAMP R package (v3.6.1) to identify DNA methylation sites with significant differences (*P* < .05). PCA algorithm was used to realize the feature reduction of all the sites contained in each gene. The first principal component was defined as module eigengenes (MEs). Further, Spearman correlation analysis was used to find the association matrix between the MEs of each module and clinical indicators event, M, N, T, and age. Among them, Spearman correlation coefficient was implemented using R function cor() in the stats R package (v3.6.1), and the method parameter was set to “Spearman.”

### 2.5. Identification and verification of key DNA methylation sites

We used MM algorithm to identify key methylation sites in m4 and m10, where the threshold of m4 module is set to |MM|>0.85 & |genetic significance|>0.17; the threshold of m10 module is set to |MM|>0.75 & |genetic significance|>0.15. In this study, survival analysis of key methylation sites was performed to identify key targets that significantly affect prognostic survival. Among them, survival analysis was implemented by R function survfit() in the survival R package (v3.6.1).^[11]^

### 2.6. Key genes differential expression analysis at multi-omics

In order to verify the reliability of key genes and investigate the regulatory relationships between different levels, firstly, the external data GSE139404, GSE48684 were used to verify the differences of key methylation sites between different tissues. Then, we applied Spearman correlation coefficient to calculate the correlation between key methylation sites and their gene expression. Finally, The Human Protein Atlas database (https://www.proteinatlas.org/) was applied to verify the differential expression of key genes at the protein level.

### 2.7. Screening potential target drugs based on molecular docking database

First, the structure of YWHAG protein was downloaded from the PDB database (https://www.rcsb.org/) and the predicted structure of MAP4 protein was downloaded from the UniProt database (https://www.uniprot.org/). Further, CTD database (https://ctdbase.org/) was applied to find target protein-related small molecule drugs, and the small molecule structures were downloaded from PubChem (https://pubchem.ncbi.nlm.nih.gov/). Finally, molecular docking was performed using Biovia Discovery Studio (2019) software. The table shows the official names and locations of the genes involved in this study (Table S7, Supplemental Digital Content, http://links.lww.com/MD/I369).

## 3. Result

### 3.1. Analysis of DMGs

In this study, samples with missing data were removed from the clinical samples. Then we obtained 84 cancer tissue samples and 6 paracancerous tissue samples with complete clinical information.

**Figure 1.**
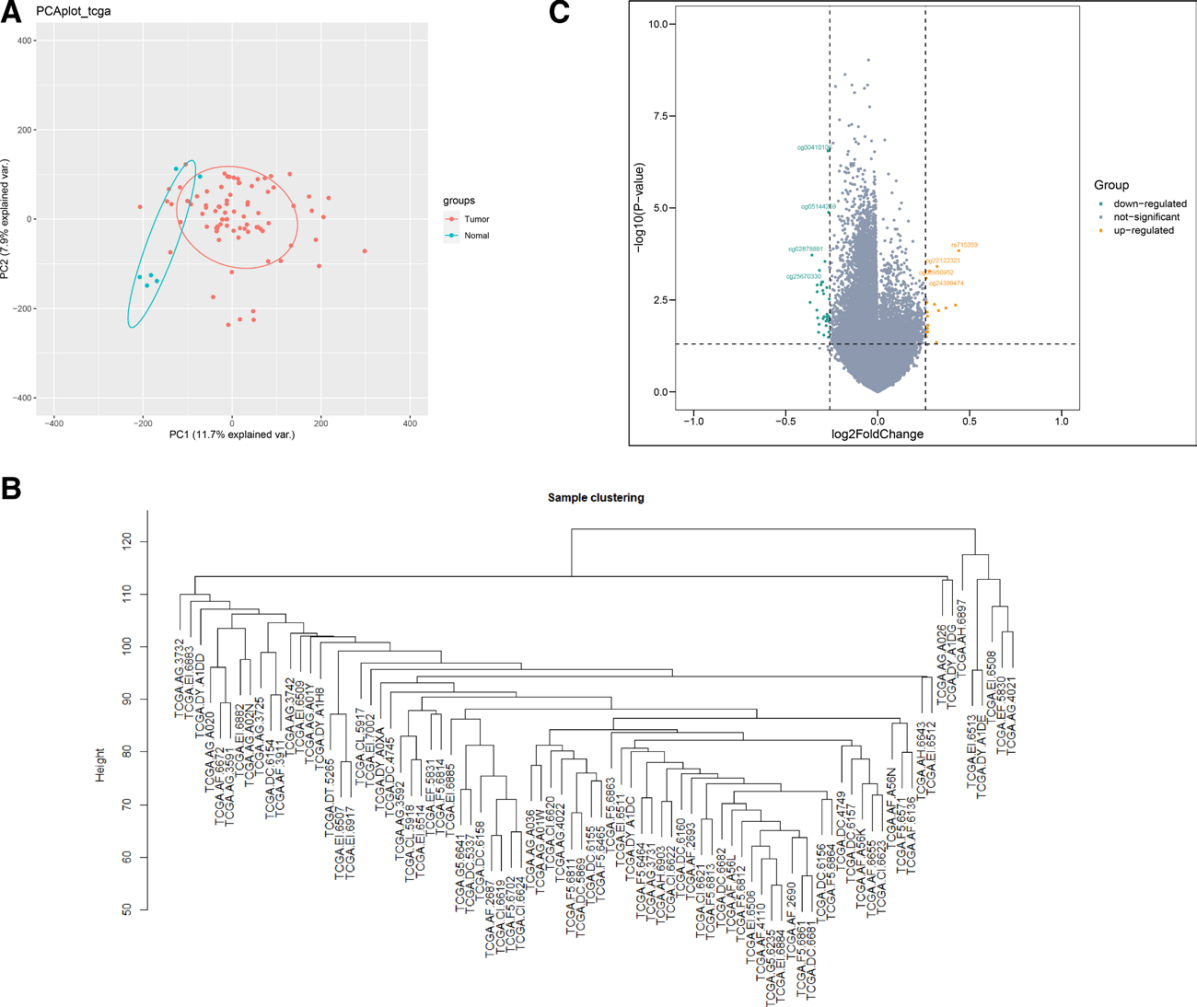
Hierarchical clustering analysis of cancer tissues methylation data. (A) Batch effect demonstration of TCGA data. (B) Results of hierarchical clustering analysis of cancer tissue samples. (C) Volcano map of differentially methylated loci. TCGA = The Cancer Genome Atlas.

Then, 6 outlier samples were found by hierarchical clustering of the cancer tissues DNA methylation profiles (Figure 1B). Then, we removed the 0 expression methylation sites and got 385,475 methylation sites (Table S1, Supplemental Digital Content, http://links.lww.com/MD/I363). The batch effect results show that there is no significant batch effect in this dataset (Figure 1A).

The final step, 5983 genes (19,064 DNA methylation sites) were used for identifying the DMGs between READ and its paracancerous tissues (Figure 1C) with champ algorithm (Table S2, Supplemental Digital Content, http://links.lww.com/MD/I364). DNA methylation sites such as rs715359, cg22122321, cg06950952, and cg24399474 are significantly upregulated and cg00410106, cg05144259, cg02878891, cg22869030, and cg25670330 are significantly downregulated.

### 3.2. Construction of weighted gene regulatory network and identification of gene modules

Genetic mutations of signal proteins can cause excessive activation of key cell signaling pathways, and epigenetic modifications of gene sequences can block the transcription of tumor suppressor genes.^[12]^ Futreal et al indicated that genes undergo methylation modifications and mutations in cancer are mainly involved in signal transduction between cells.^[13]^ CCA was used to calculate the correlation between genes at the methylation level as the weight of the relationship between genes, a large network including 4719 genes and several small networks with fewer than 10 genes were built. We defined the large network as the weight gene regulatory network (Table S3, Supplemental Digital Content, http://links.lww.com/MD/I365).

**Figure 2.**
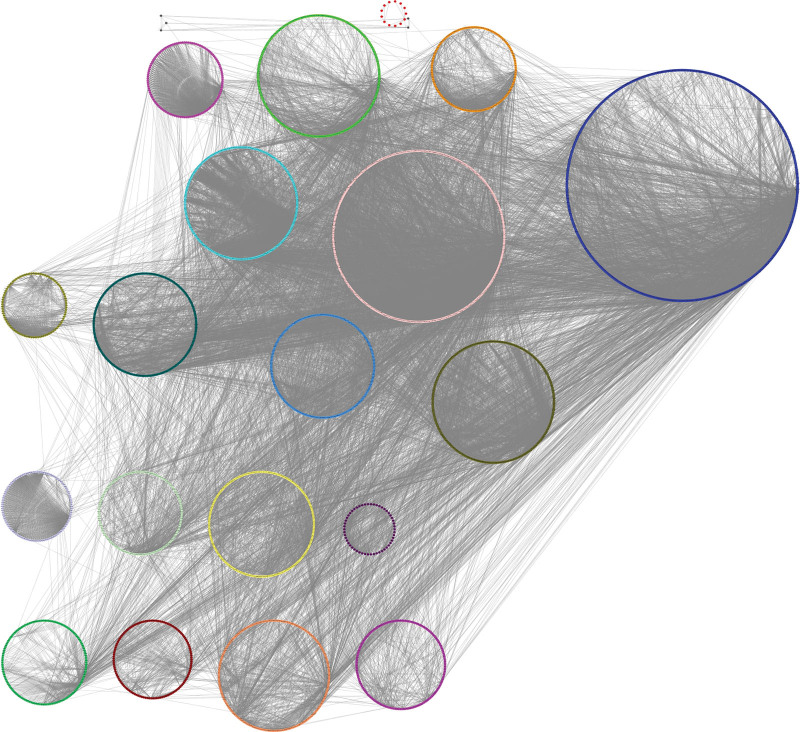
Division result obtained by multilevel algorithm.

To identify gene modules in the network, multilevel algorithm was used to divide the weighted gene regulatory network into 20 gene modules (modularity = 0.59), removed gene modules with <100 genes, and obtained 16 gene modules (Figure 2).

### 3.3. Association analysis between gene modules and clinical information

**Figure 3.**
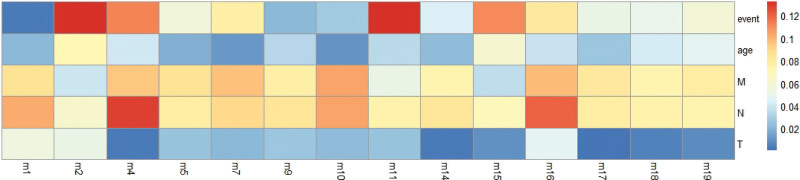
The heatmap of gene modules and clinical characteristics.

To figure out the potential connection between gene modules and clinical phenotypes, background data was cancer tissue methylation data with statistically significant methylation sites (*P* < .05). The first principal component of each module which was defined as MEs by PCA algorithm. And then, we used Spearman correlation analysis to calculate the correlation between clinical features event, age, M, N, T, and MEs (Figure 3). Among them, T, N, and M refer to the condition of the primary tumor, the involvement of regional lymph nodes, and distant transfers, respectively.^[14]^

The results showed as in Figure 3: Characteristics event and age were highly associated with module 2; Characteristic M was highly associated with module 10; Characteristic N was highly associated with module 4; and Characteristic T was highly associated with module 1.

### 3.4. Gene ontology (GO) enrichment analysis of gene modules

From results we got, modules 1, 2, 4, and 10 were found significantly correlated to clinical characteristics. We used GO enrichment analysis to explore biological significance of these modules (Table S4, Supplemental Digital Content, http://links.lww.com/MD/I366).

**Figure 4.**
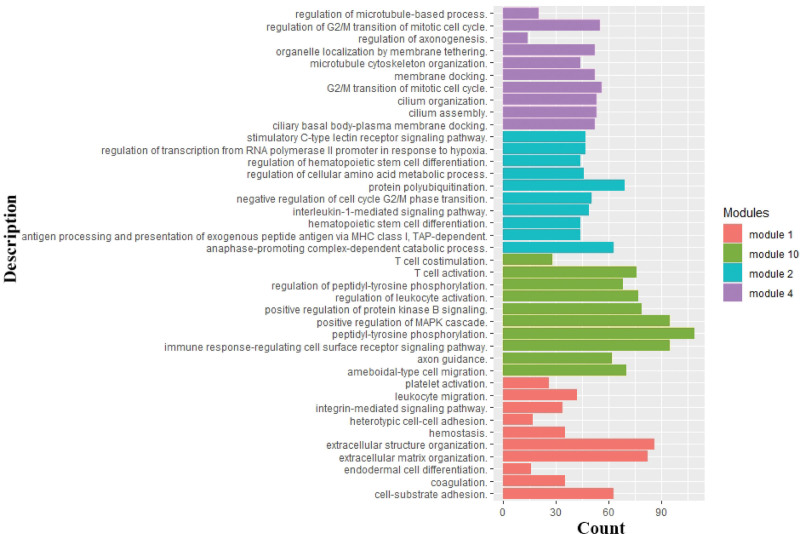
The GO enrichment results of modules. GO = gene ontology.

Module 1 highly related to characteristic T (*R* = 0.078) participated in the regulation of extracellular matrix and microfilament formation. Module 2 highly associated with characteristic event (*R* = 0.161) and age (*R* = 0.117) participated in the regulation of cell metabolism, cell polarity, and cell cycle. Module 4 highly related to characteristic N (*R* = 0.165) participated in the regulation of regulation of cytoskeleton formation with microtubules and flagella, synapse formation and mitosis. Module 10 highly associated with characteristic M (*R* = 0.137) participated in fibroblast growth factor chemotaxis and immune cell activation regulation, and cell migration. Top 10 biological processes sorted by logP for each module were shown in Figure 4.

### 3.5. Identification of key DNA methylation sites

Characteristic M, N was important indicators for tumor metastasis evaluation. In this study, modules 4 and 10 closely related to tumor metastasis were selected for further research.

To explore the key DNA methylation sites in modules, we performed MM algorithm to screen key methylation sites in modules m4 and m10 (Table S5, Supplemental Digital Content, http://links.lww.com/MD/I367). Among them, key DNA methylation sites in module 4 were cg04441191 (MAP4), cg05658717 (KSR2), cg09622330 (GRIN2A), cg10698404 (YWHAG), cg17047993 (SPAG9), cg17917959 (AZI1), cg22055790 (CLASP1), cg24504843 (CEP135), cg24531267 (CEP250), and cg26231243 (PIN1).

Key DNA methylation sites in module 10 were cg02658564(HGFAC), cg08432013(DLG2), cg26449294(DLG2), cg09319815(PDCD1), cg10526431(PDCD1), cg14842771(EPHB6), cg15499275(PIK3CD), cg16376000(FGF12), cg22325673(PPP2R2C), and cg23852348(PDGFD).

### 3.6. Survival analysis of key DNA methylation sites

**Figure 5.**
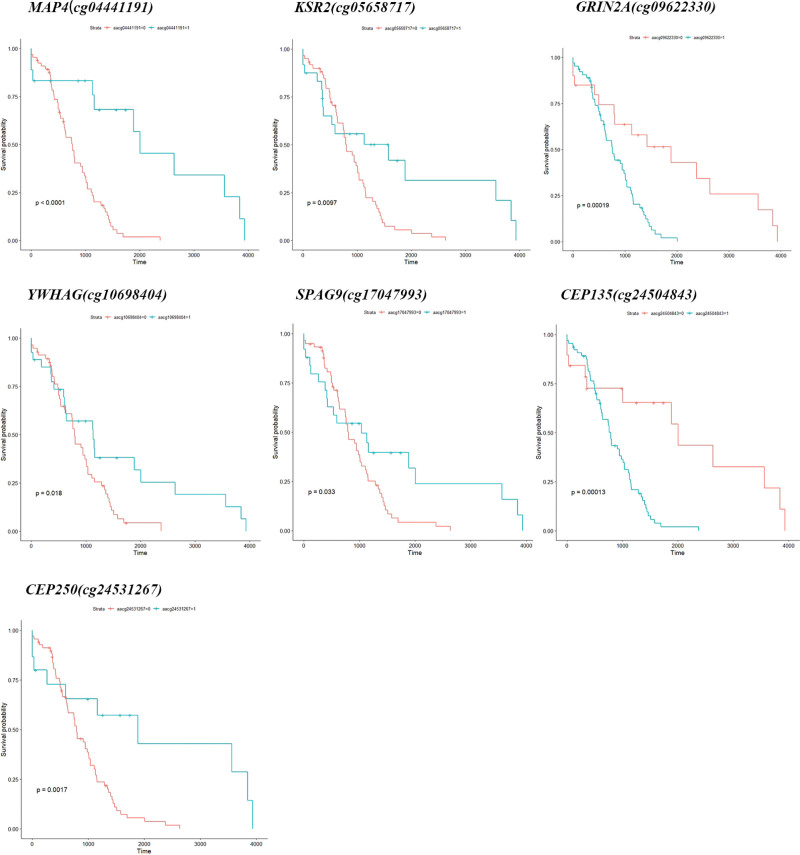
Survival analysis of key DNA methylation sites. DNA = deoxyribonucleic acid.

To identify prognostic targets which affect the prognostic survival status of READ, survival analysis of key DNA methylation sites indicated that cg04441191 (MAP4), cg05658717 (KSR2), cg09622330 (GRIN2A), cg10698404 (YWHAG), cg17047993 (SPAG9), cg24504843 (CEP135), and cg24531267 (CEP250) were closely related to the prognostic survival (Figure 5).

### 3.7. Key methylation sites validation and multi-omics exploration of key gene regulatory mechanisms

**Figure 6.**
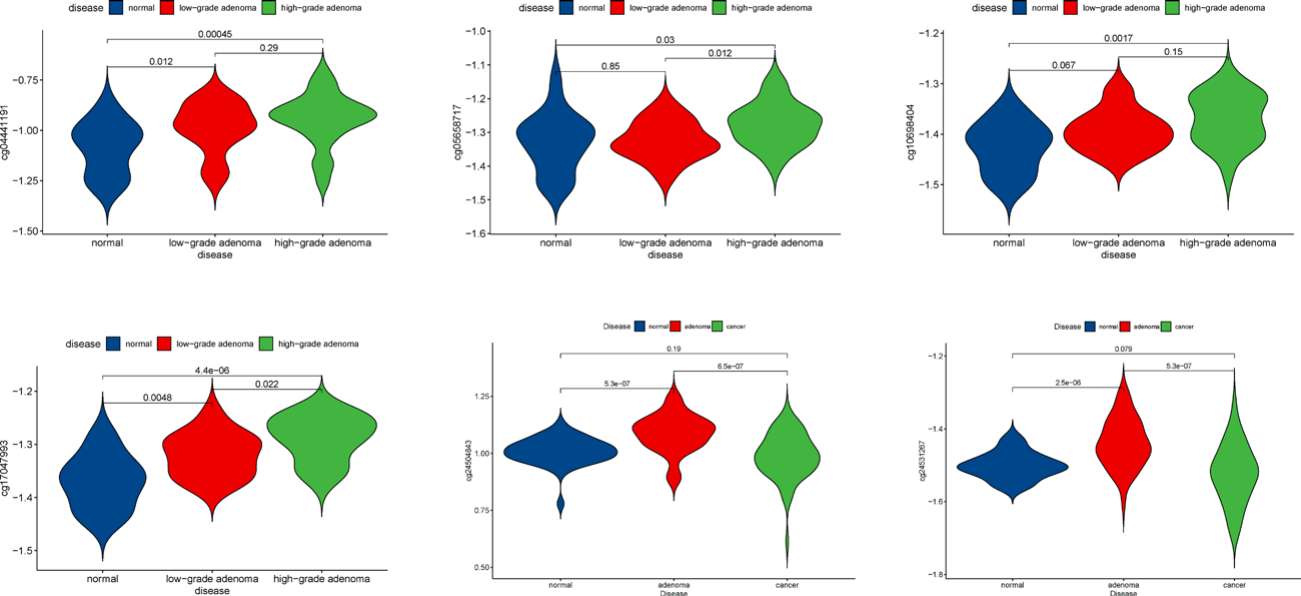
Validation of differentially methylated loci.

**Figure 7.**
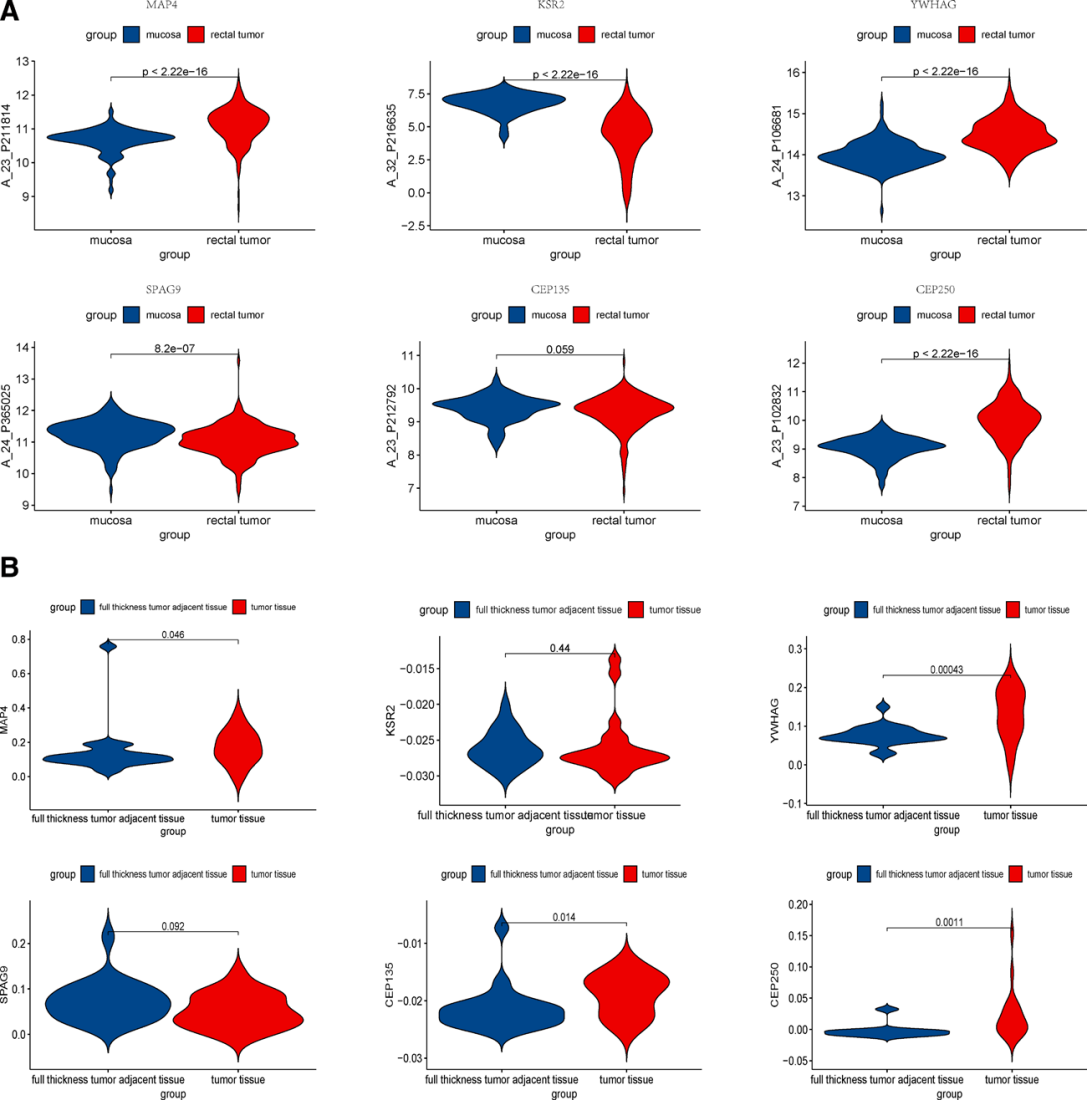
Differences in DNA methylation sites-associated genes at the mutational, transcriptional levels. (A) Differences in gene-related mutation loci. (B) Differential expression of related genes at the transcriptional level. DNA = deoxyribonucleic acid.

**Figure 8.**
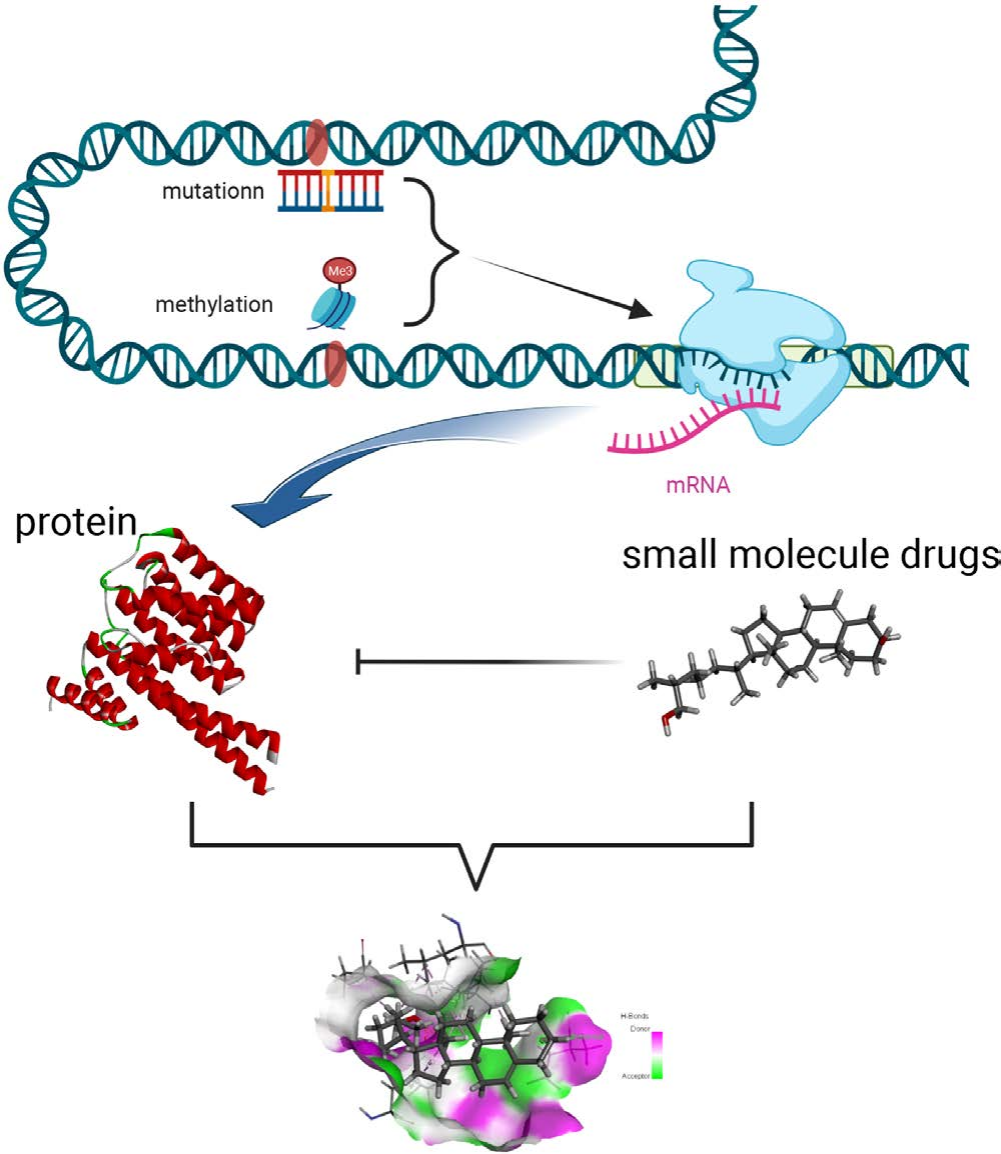
Immunohistochemistry and molecular docking of key genes. (A) Immunohistochemical staining of YWHAG protein in normal and tumor tissues. (B) Immunohistochemical staining of MAP4 protein in normal and tumor tissues. (C) Molecular docking of YWHAG proteins with 27-hydroxycholesterol compounds simulates in vivo interactions. (D) Molecular docking of MAP4 proteins with artenimol compounds simulates in vivo interactions. MAP4 = microtubule-associated protein 4.

To identify the significant differences in methylation sites between cancer and precancerous lesions, the GEO datasets GSE139404 and GSE48684 were selected to validate the key methylation sites, and we found that the methylation of cg04441191 (MAP4), cg05658717(KSR2), cg10698404 (YWHAG), cg17047993 (SPAG9), cg24504843 (CEP135), and cg24531267 (CEP250) sites were significantly different between normal and tumor tissues (Figure 6). Next, to investigate the effect of DNA methylation on downstream transcriptional expression, we applied Spearman correlation to calculate the association between DNA methylation *β* values and gene expression in the same cancer sample and found strong correlations between DNA methylation and transcription in MAP4, YWHAG, KSR2, SPAG9, and CEP135 (Table S6, Supplemental Digital Content, http://links.lww.com/MD/I368). The trend was the same as Cheng et al.^[15]^ In addition, we found that the variability of methylation sites was more significant in normal colorectal tissues versus the adenoma group than versus the cancer group (Sub-Figure 1). At the single nucleotide polymorphism (SNP) mutation level and transcriptional level, we found that MAP4, YWHAG, KSR2, SPAG9, and CEP250 gene-related loci are different in cancer and paracancer tissues, and MAP4, YWHAG, CEP135, and CEP250 differed significantly at the transcriptional level (Fig. 7). Considering DNA methylation, SNP and transcriptional levels, we took the intersection to obtain YWHAG2 and MAP4 as key genes and analyzed the protein level from The Human Protein Atlas database and found that YWHAG was medium expressed in endothelial cells and glandular cells, and highly expressed in cancer tissues. The expression of MAP4 was high in endothelial cells, medium in glandular cells and high in cancerous tissues (Fig. 8A and B).

**Figure 9.**
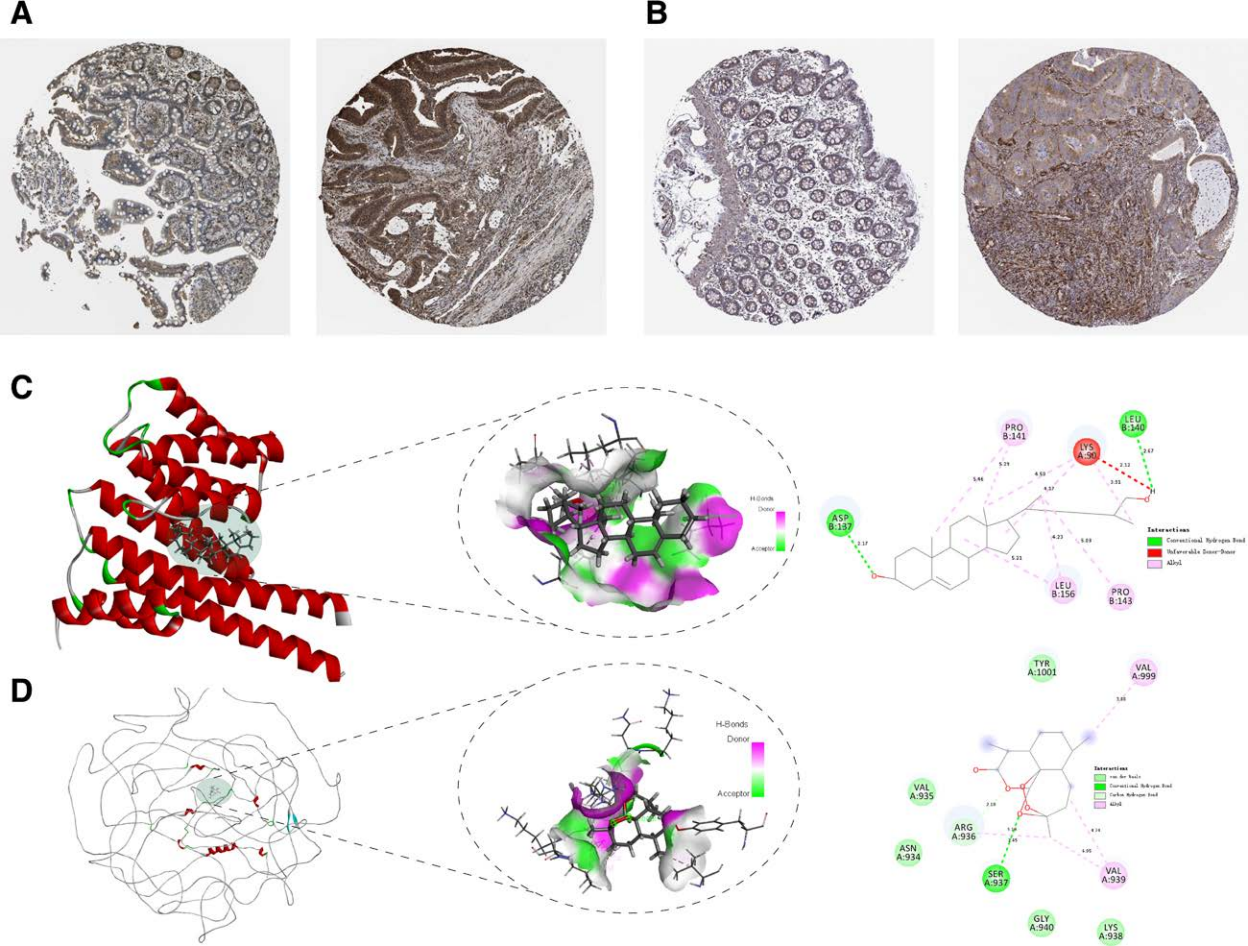
Mechanism of DNA methylation and SNP mutations affecting translation. DNA = deoxyribonucleic acid, SNP = single nucleotide polymorphism.

In summary, this study infers that SNP mutations and DNA methylation jointly affect gene transcription and regulate gene translation (Fig. 9).

### 3.8. Potential targeted drug identification and molecular docking

In order to screen the potential target drugs for 2 target proteins, this study applied the CTD database to predict potential drugs of 2 proteins and obtain artenimol as a target drug for MAP4 protein and 27-hydroxycholesterol as a target drug for YWHAG protein. To predict the binding sites between small molecules and proteins, we downloaded the YWHAG protein structure from the PDB website and MAP4 protein predicted structure through the UniProt website. We found that Artenimol formed a conventional hydrogen bond with the A subunit, 5 van der waals and 1 carbon hydrogen bond with the MAP4 active site. 27-Hydroxycholesterol formed 2 pairs of conventional hydrogen bonds with YWHAG in the B subunit and 1 pair of unfavorable donor-donor with A subunit in the protein active site (Figure 8C and D).

## 4. Discussion

CRC has become the second leading cause of death in the US, threatening people’s health, and 30% CRC cases are READ cases.^[16]^ Therefore, this study on the mechanism of READ at the methylation level can provide a key role for the diagnosis and early treatment of READ. We proposed a bioinformatics method based on weighted network analysis to analyze the pathological mechanism of READ at DNA methylation level, including analysis of DMGs, construction of weighted gene regulatory network, identification of gene modules, identification of key DNA methylation sites. Besides, DNA methylation sites were validated, then explored the mechanisms of key genes regulated at the DNA methylation, SNP, transcription, and protein levels and searched for potential target therapeutics at the protein level.

During algorithm design, considering that each gene contains multiple DNA methylation sites, and the number of methylation sites were different among genes. Thus, algorithms for evaluating the correlation of a single variable represented by Pearson correlation analysis and Spearman correlation analysis etc can’t satisfy the description of the correlation of feature vectors of different dimensions. CCA was an evaluation algorithm that describes the correlation between vectors of different dimensions. Thus, we chose CCA algorithm to calculate the correlation of genes at the DNA methylation level. CCA algorithm was more accurate in evaluating data whose number of features was less than the number of samples.^[17]^ To this end, PCA algorithm was used to perform feature reduction on the data of each gene methylation site, ensuring the CCA results more accurate. In terms of correlation analysis between clinical features and gene modules, we have selected statistically significant methylation sites corresponding to each gene module for subsequent analysis, avoiding the noise signal generated by all site information of each gene, and ensuring more accurate identification of key sites.

From GO enrichment results, it was found that characteristic M was related to biological processes such as the chemotaxis of fibroblast growth factors and the activation and regulation of immune cells etc.; characteristic N was associated with the regulation of cytoskeleton formation, mainly microtubules and flagella, the regulation of synapses, and the regulation of cell mitosis. Fibroblast growth factor mainly regulates cell proliferation, migration and differentiation and is closely related to tumor development.^[18]^ Actin and tubulin combined to form a highly versatile dynamic polymer, which could construct cytoplasmic organelles and intracellular compartments, defined cell polarity and generate thrust and contractility to affect tumor proliferation and metastasis.^[19]^ In this respect, we analyzed these modules highly related to characteristic M and N, and found that: DNA methylation of MAP4, KSR2, GRIN2A, YWHAG, SPAG9, CEP135, CEP250 was closely related to the prognostic survival. From the results of external dataset validation, we found that the methylation levels of the above loci were more closely related to the grade of adenoma, which is thought to be significantly correlated with cancer. We therefore hypothesize that methylation is involved in and influences the differentiation process of tissues from hypomethylation status at onset to hypermethylation status in adenoma to hypomethylation status in cancer, and DNA methylation may serve as a potential dynamic marker for stepwise changes in disease.^[20]^ The effect of DNA methylation may require regulation of transcriptional and post-translational protein expression to achieve an effect on a biological function. For this reason, we did a correlation analysis between DNA methylation and transcriptional levels, and considering the possibility that mutations may have the same consequences, we chose to take intersections to find genes that differ and are associated with transcriptional as well as protein levels (YWHAG and MAP4) as the CTD database identified artenimol and 27-hydroxycholesterol as potential targeting agents for READ. It is worth mentioning that both proteins were found to be expressed in normal colon tissues and colon cancer tissues in this study. However, the protein expression of glandular cells in normal tissues differed from that of cancer tissues, and their expression differed from that of their own endothelial cells. We speculate that increased methylation of endothelial cells leads to changes in the normal tissue microenvironment.^[21]^ Development of adenoma and continued progression of adenoma to cancer driven by a continued increase in hypermethylation status, with progression to cancer status followed by a reduction in methylation levels to a new equilibrium.^[20]^

MAP4 was a member of microtubule-associated protein, which could combine with microtubules to form microtubule side arms. Microtuble side arms had the function of stabilizing the spatial structure of the microtubule and promoting the aggregation of the microtubule into a bundle.^[22]^ Ibtissem Nabti et al suggested that tau-associated MAP4 could bias the bidirectional transport of organelles toward the negative end of microtubules. This coincides with MAP4 phosphorylation mediated by the kinase GSK3β, and MAP4-dependent redistribution of organelles may be prevalent in cancer cells.^[23]^ Chen et al found that MAP4 affected the migration of epidermal cells by regulating the expression of Tctex-1.^[24]^ In the occurrence and development of tumors, Xia et al found knockdown MAP4 could effectively inhibit the migration and invasion of lung adenocarcinoma cells, and the expression of MAP4 could be used as an independent indicator of prognostic survival of lung adenocarcinoma.^[25]^ MAP4 is hypermethylated in prostate cancer.^[26]^ Amino acid mutations in MAP4 protein reduce the sensitivity of vincristine in the treatment of leukemia.^[27]^ Hypermutated and hypermethylated MAP4 is associated with poor prognosis in gliomas.^[28]^ Ou et al found that the cAMP/PKA signaling pathway may inhibit the invasion of bladder cancer cells through targeted regulation of MAP4 phosphorylation.^[29]^ MAP4 is widely involved in PI3K/AKT pathway in kidney injury^[30]^ as well as in lumbar disc herniation-related diseases.^[31]^

YWHAG was mainly involved in the regulation of mitosis and the signal transduction between cells and other biological processes, and was closely related to tumor migration and proliferation.^[32]^ Wang et al found that the upregulation of YWHAG could inhibit the viability, proliferation, migration, invasion, and mitosis of glioblastoma.^[33]^ Chu et al found that miR-222 could inhibit the proliferation and invasion of osteosarcoma cells by downregulating the expression of YWHAG.^[34]^

Hypermethylated YWHAG is thought to be associated with Parkinson,^[35]^ while YWHAG mutation-related studies are mainly in epilepsy and neurobehavior.^[36,37]^ However, Ni, J., et al found that haplotype mutations significantly increased enhancer activity and YWHAG expression. The expression levels of YWHAG genes were higher in all gastric cancer tissues than in the paracancerous tissues, suggesting a poor prognosis for gastric cancer patients. Genes co-expressed with YWHAG showed abundant expression in the RAS signaling pathway.^[38]^ In pancreatic cancer, CERS6-AS1 increases YWHAG expression by sponging miR-217-5p (miR-217), leading to phosphorylation of RAF1 and activation of ERK signaling,^[39]^ and YWHAG is also involved in the regulation of PI3K/AKT pathway.^[40]^

In summary, we performed the bioinformatics analysis method based on weighted network analysis to analyze the methylation level of READ. It was found that DNA methylation sites of 7 genes such as MAP4 (cg04441191), KSR2 (cg05658717), GRIN2A (cg09622330), YWHAG (cg10698404), SPAG9 (cg17047993), CEP135 (cg24504843), and CEP250 (cg24531267) were closely related to the prognosis of READ, which could be used as prognostic markers of READ. These genes have been more or less reported to be related to invasion and metastasis of tumor and as the key biomarkers for detection or prognostic diagnosis. In addition, it was found that these biological processes such as regulation of fibroblast growth factor chemotaxis, immune cell activation as well as regulation of cytoskeleton formation with microtubules and flagella, regulation of synapse, and regulation of cell mitosis played key roles in the development of READ. Based on the above results, we explored the potential molecular mechanism of READ from the methylation level, including the relationship between DNA methylation and gene transcription. We propose a possible mechanism: mutations and DNA methylation affect gene transcription and regulate translation. Finally, we screened potential targeting drugs against DNA methylation-related proteins to hinder the progression of adenoma to cancer and improve patient prognosis providing new insights for the diagnosis and treatment of READ.

## Author contributions

**Conceptualization:** Xiaoqiang Pan, Xingcheng Yi, Maozhuo Lan, Xiaoyun Su, Fang Zhou, Wei Wu.

**Data curation:** Xingcheng Yi.

**Formal analysis:** Maozhuo Lan, Wei Wu.

**Investigation:** Xiaoqiang Pan.

**Methodology:** Xiaoqiang Pan, Maozhuo Lan, Wei Wu.

**Software:** Xiaoyun Su, Fang Zhou.

**Validation:** Xiaoqiang Pan.

**Visualization:** Xiaoqiang Pan.

**Writing – original draft:** Xiaoqiang Pan, Xingcheng Yi, Xiaoyun Su, Wei Wu.

**Writing – review & editing:** Xiaoyun Su, Wei Wu.

## Supplementary Material
